# Does the use of patient-specific instrumentation improve resource use in the operating room and outcome after total knee arthroplasty?–A multicenter study

**DOI:** 10.1371/journal.pone.0277464

**Published:** 2022-11-11

**Authors:** Franziska Beyer, Cornelia Lützner, Michael Stalp, Georg Köster, Jörg Lützner

**Affiliations:** 1 University Center of Orthopaedic, Trauma and Plastic Surgery, University Hospital Carl Gustav Carus, TU Dresden, Dresden, Germany; 2 Department for Orthopaedic and Trauma Surgery, Helios Kliniken Mittelweser, Nienburg/Weser, Germany; 3 Department of Orthopaedic Surgery, Schön Klinik Lorsch, Lorsch, Germany; Assiut University Faculty of Medicine, EGYPT

## Abstract

Patient-specific instrumentation (PSI) in total knee arthroplasty (TKA) has been introduced to reduce instruments and surgical time and to improve implant alignment. The aim of this study was to compare TKA with patient-specific and conventional instrumentation with regard to the use of resources in the operating room (OR), alignment and patient-reported outcome. A total of 139 TKA with PSI or conventional instrumentation were included in three centers. Economic variables of the surgery (number of instrument trays, setup and cut-sew-time), radiological alignment and patient reported outcomes (VAS Pain Scale, Oxford Knee Score, EQ-5D) were assessed after 6 weeks, 6 and 12 months. There was a significant reduction of instrument trays and of time in the OR in the PSI group. The reduction varied between the centers. With strict reorganization, more than 50% of the instrument trays could be reduced while using PSI. There were no significant differences in cut-sew-time, implant position, leg axis, pain and function. The use of PSI was associated with significantly less OR resources. However, the savings did not compensate the costs for this technology.

## Introduction

Total knee arthroplasty (TKA) is an efficient and cost effective treatment option for advanced osteoarthritis of the knee. Despite its general success, there is a relevant number of about 20% of patients who remain unsatisfied after TKA. Many reasons have been identified which contribute to unsatisfactory results following TKA including pain and function, adverse events, unfulfilled expectations but also surgical and implant-related factors. Alignment is generally thought to be a critical factor for good results and TKA survival. Malalignment by itself or by consecutive wear and loosening is still an important cause for revision [1].

Several technologies have been introduced to improve alignment. Computer-assisted surgical navigation (CAS) has been proven to improve accuracy and precision of implant and leg alignment in TKA [2]. However, there are still barriers for general use of CAS which include additional time, additional equipment in the operating room (OR) and costs. To address all these drawbacks patient-specific instrumentation (PSI) has been introduced. PSI uses magnetic resonance imaging (MRI) or computed tomography (CT) of the hip, knee and ankle joint to create a three-dimensional (3D) model of the knee including the leg axis. This 3D model is used for preoperative planning of implant position and size. Based on this planning patient-specific cutting guides are produced. These cutting guides are designed to fit in exactly one position and substitute therefore conventional alignment rods [3]. Theoretical advantages include less instruments in the OR, reduced surgical time, reduced blood loss and improved implant alignment. Therefore, despite additional imaging and the production costs of the cutting-guides, this technology could be cost-efficient. While many studies have been published, which focused on radiographic outcomes, only some studies [4–6] investigated patient-reported outcome (PRO) and few analyzed economical aspects [7–11], none of them within the German healthcare system.

The aim of this study was to compare TKA with patient-specific and conventional instrumentation with regard to use of resources in the OR, alignment and PRO. We hypothesized less resource use in the OR, better alignment and similar PRO with PSI.

## Materials and methods

**Fig 1:**
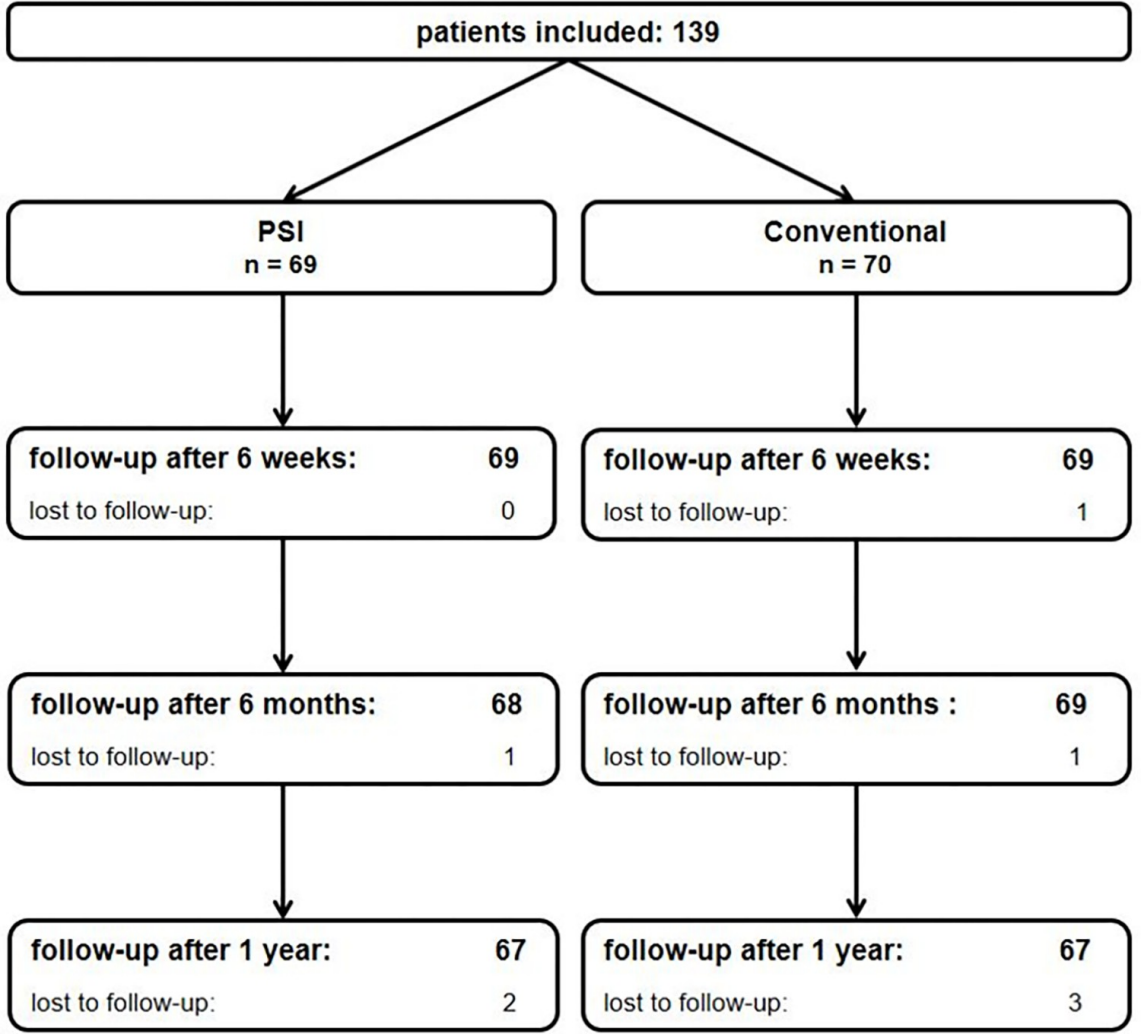
Flow-chart of included patients.

The study has been performed in compliance with the Helsinki Declaration and has been approved by the ethics committee of the TU Dresden (EK 427122011). All patients signed informed consent. Between July 2012 and February 2016 patients with osteoarthritis of the knee, which were scheduled for TKA in three arthroplasty centers in Germany, were screened. Exclusion criteria included a severe osteoarthritis of the hip, stiffness of the hip or knee, neurological disorders, revision TKA, any metal implant close to the knee, which would influence the quality of the MRI, and any arterial disease that would exclude the use of a tourniquet. A total of 139 patients were included (Fig 1). Patients were evaluated before surgery, six weeks, six months and one year after surgery. Patients underwent physical examination and were asked to fill in the Oxford Knee Score [12], the EuroQol (EQ-5D 3L) [13] and a Pain Visual Analog Scale (VAS) [14]. A whole-leg standing radiograph was performed before surgery and at the six-months follow-up. An independent investigator (CL) who was not involved in the surgeries or follow-up visits reviewed and measured all x-rays with the use of mediCAD software (mediCAD HECTEC, Landshut, Germany). Data of 134 (96%) patients were obtained, one patient was revised and 4 patients refused further participation (Fig 1). For these patients without follow-up, only the surgical data was included in the analysis.

**Fig 2:**
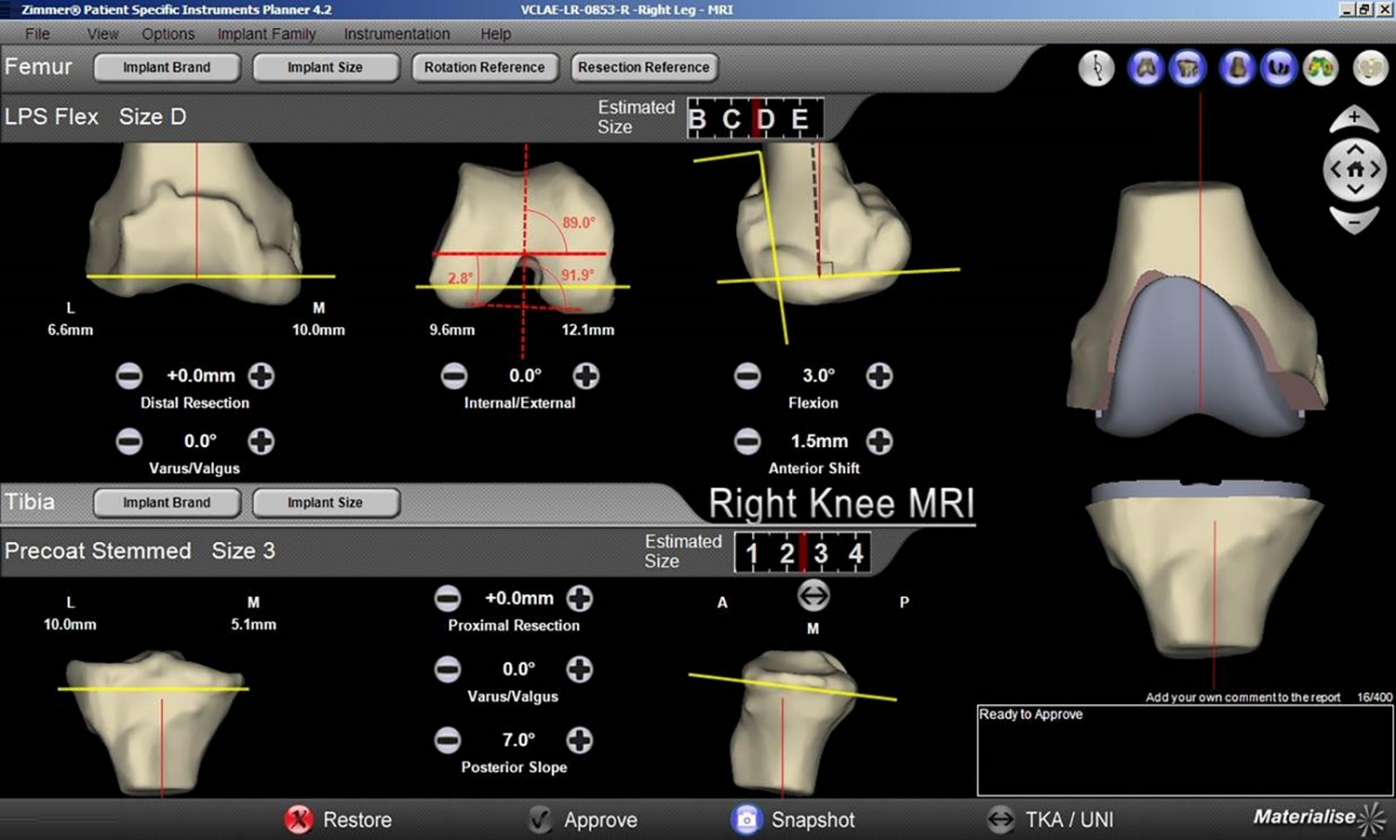
3D planning of the component position which can be adjusted by the surgeon as needed.

In each center, patients were operated consecutively with conventional and patient-specific instrumentation using Zimmer NexGen Solutions (Zimmer Biomet, Warsaw, IN). All surgeries were performed by experienced arthroplasty surgeons with equivalent experience in knee arthroplasty. Patients with conventional instrumentation served as control group and were operated according to the standard of each center. All centers used a tourniquet, a medial parapatellar approach and all components were fixed with bone cement. Patellar resurfacing was not performed. In all patients a neutral leg axis was planned. Patients of the PSI group received an MRI including the hip, knee and ankle joint before surgery. The images were uploaded to Materialise (Materialise B.V., Leuven, Belgium) for processing and creation of a 3D model. Then surgical planning was performed by the operating surgeon using the Materialise planning software (Fig 2) and after the surgeon’s approval the PSI cutting guides were produced by Materialise and sent to the center. All surgeons had sufficient training with the PSI technology and made five surgeries with the PSI tools before recruiting patients. During surgery, several economic outcomes were collected including number of instrument trays, setup time and cut-sew time. In the PSI group additional information regarding the cutting guides were assessed, e.g. problems with fitting of the cutting guides, adjustments of resections and surgeon’s satisfaction with the patient specific instrumentation (measured on a Likert-Scale with answers “very unsatisfied“, “somewhat unsatisfied“, “satisfied” and “very satisfied”).

Additional and saved costs of PSI were calculated using German hospital estimates. In Germany PSI instrumentation is associated with additional costs of approximately 300 € for the preoperative MRI and 450 € for the production of the cutting guides. Costs of 65 € for processing and sterilization per instrument tray and 36 € per minute in the OR were used to calculate savings in the OR.

The sample size was calculated to detect a minimal clinical relevant difference of 5 minutes in the OR with a standard deviation of 10 minutes. With a power 0.8 and a significance level of p < 0.05 a total of 64 patients per group were necessary. To account for a drop-out of 10%, a minimum of 72 patients were included in each study arm.

All data was entered into a web-based CRF. After completion of the study, data was then exported into SPSS for analysis (IBM Corp, Armonk, NY). Data description was based on means and standard deviation for continuous variables and medians and percentiles for categorical variables. Comparisons between the conventional and PSI groups were performed using t-tests and analysis of variance for continuous variables and chi-square tests for categorical variables. All analyses have been performed with SPSS version 23 statistical software.

## Results

**Fig 3:**
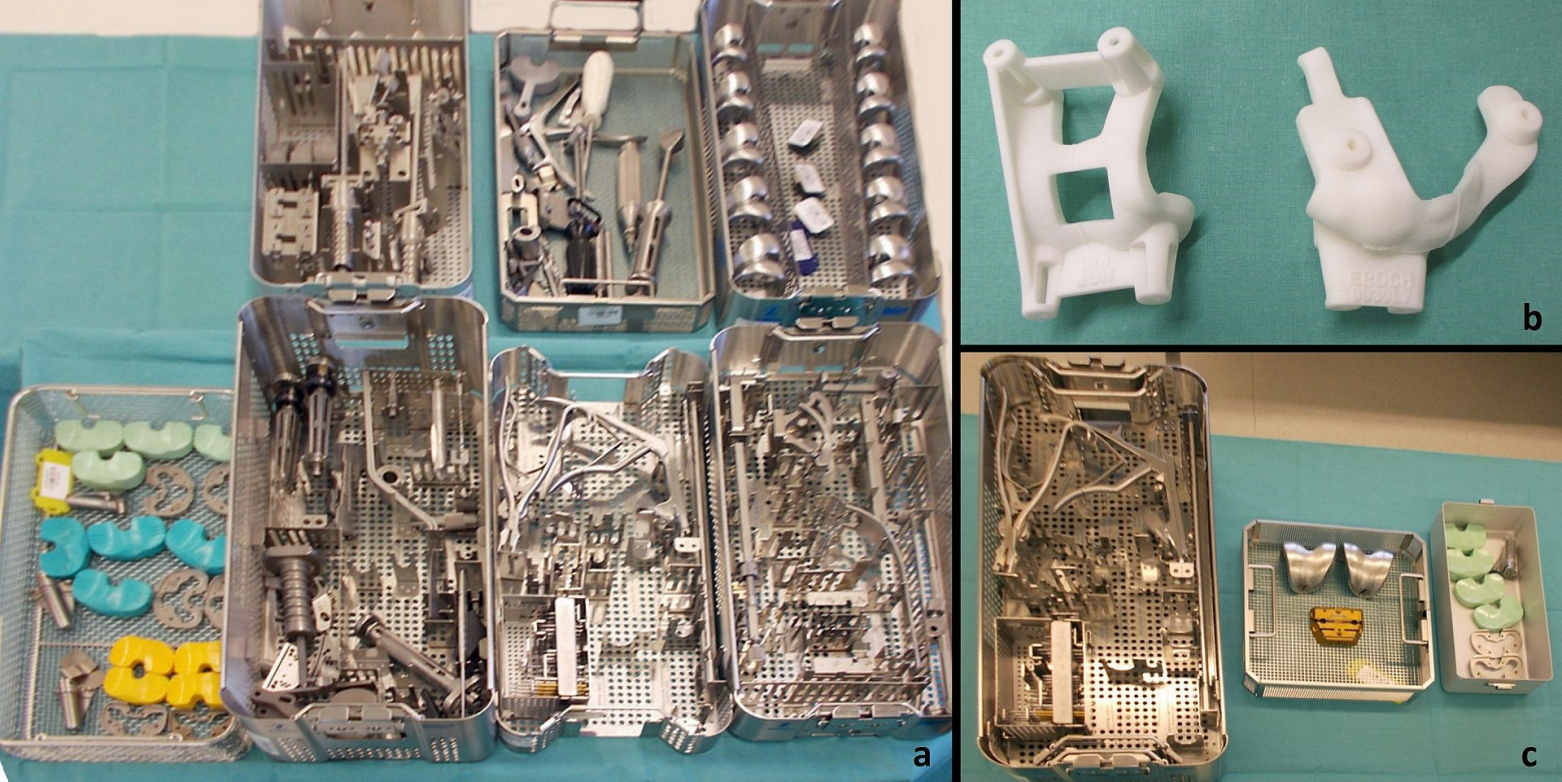
Instrumentation for conventional TKA (a), Patient-specific cutting-guides (b) and reduced Instruments for PSI TKA (c).

**Fig 4:**
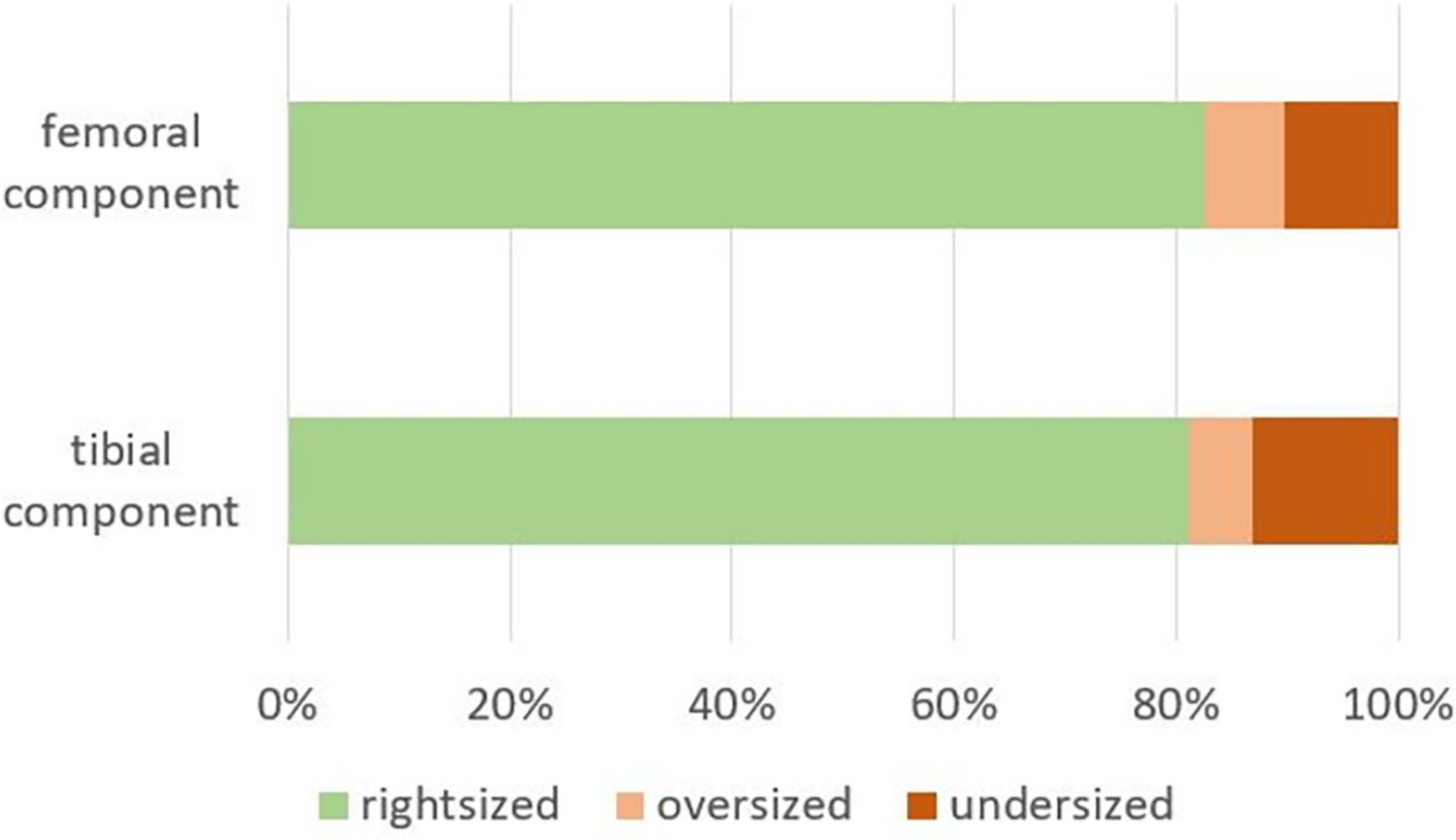
Relative frequencies of divergence to PSI planned sizing of the femoral and the tibial components.

No differences were found in age or gender between the groups (Table 1). The patients in the PSI group had a slightly lower body mass index (BMI) compared to the conventional group (28.9 vs 30.9, p = 0,027). OR setup-time, tourniquet time and cut-sew time were not different between the groups. The total time in the OR (setup-time + cut-sew-time) was significantly lower in the PSI group (mean 91.4 vs 97.1 minutes, p = 0.035) Overall the number of instrument trays was significantly lower in the PSI group compared to the conventional group (median 6 vs 7 trays, p<0.001). However, there were large variations between the centers (Table 1, Fig 3). The surgeon was very satisfied with the patient specific cutting guides in 63.8%, satisfied in 30.4% and somewhat satisfied in 5.8%. There were problems achieving a stable position of the cutting guide in only one case each on the femur and the tibia. Resections were performed as indicated in 60.9% and adjustments were made in 39.1%, mainly resection height of the tibia was adjusted but in one case, also rotational orientation of the femur was changed. Additional instruments from the standard instrumentation trays were needed in 10.1%. In two cases the femoral cutting block did not fit at all and manual instrumentation was used instead. The final sizing was as planned in 82.6% of the femoral and 81.2% of the tibial components (Table 2, Fig 4).

**Table 1:** Demographic and surgical data.

	PSI N = 67	Conventional N = 67	p-value
**Gender (% female)**	38 (56.7%)	44 (65.7%)	0.287
**Age (years)**	69.6 (8.4)	68.3 (8.7)	0.390
**BMI (kg/m** ^**2**^ **)**	28.9 (4.5)	30.9 (5.8)	0.027
**# Instrument Trays (overall)**	6 (3, 6)	7 (6, 9)	<0.001
**# Instrument Trays (center 1)**	6 (6;6)	8 (8;8)	<0.001
**# Instrument Trays (center 2)**	3 (3;5)	7 (7;9)	<0.001
**# Instrument Trays (center 3)**	6 (3;6)	6 (6;6)	0.069
**OR Setup Time (min)**	30.0 (12.5)	32.1 (14.9)	0.374
**Cut-sew Time (min)**	61.3 (13.7)	65.0 (12.5)	0.111
**Total OR Time min** *	91.4 (13.7)	97.1 (17.3)	0.035
**OR Time (center 1)**	84.7 (11.5)	83.3 (7.7)	0.618
**OR Time (center 2)**	96.1 (14.2)	105.3 (14.8)	0.036
**OR Time (center 3)**	94.0 (12.9)	104.4 (18.7)	0.049
**Tourniquet Time (min)**	60.4 (14.2)	61.3 (15.4)	0.736

* Total OR Time = OR Setup Time + Cut-sew Time

Data given as mean (SD) for continuous values, absolute (relative) frequencies for gender and median (minimum, maximum) for the number of instrument trays

**Table 2:** Required adjustments when using PSI.

	Femoral	Tibial
**Final size equal to planned size**	57 (82.6%)	56 (81.2%)
**Difficulty achieving stable resting position of the cutting guide**	1 (1.4%)	1 (1.4%)
**Orientation/Resection adjustments**	27 (39.1%)	
**Additional instruments used during the surgery**	7 (10.1%)	

Data given as absolute (relative) frequencies

The implant had to be revised in one patient in the conventional group due to tibial loosening after six months. In one patient in the conventional group an infection occurred, which was successfully treated with irrigation, debridement and inlay exchange. Manipulation under anesthesia was necessary in two patients in the PSI group.

**Fig 5:**
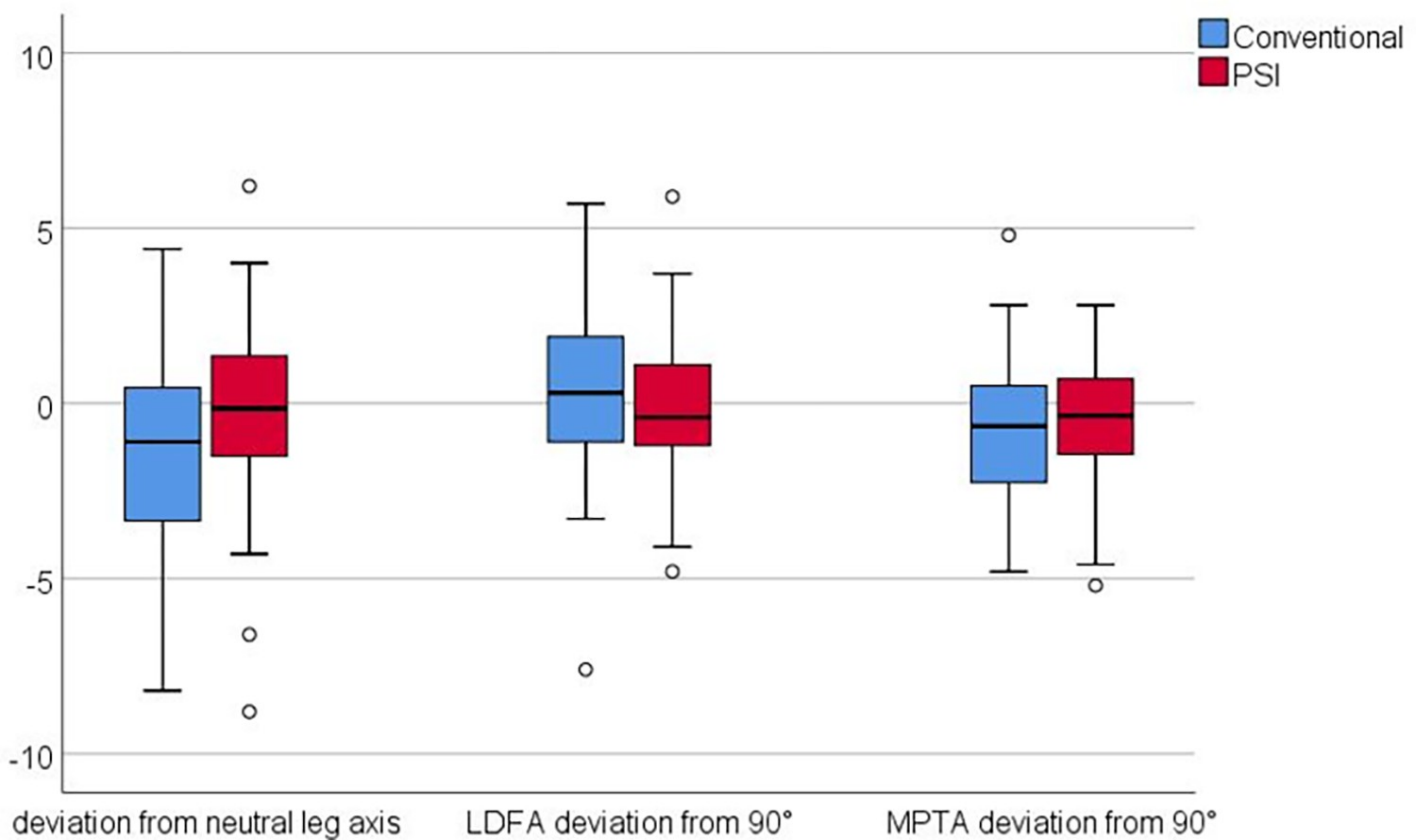
Alignment of the leg, the femoral and tibial component in the PSI and conventional group. The box represents 25^th^ to 75^th^ percentiles, negative values correspond to varus deviation, positive values correspond to valgus deviation, LDFA–lateral distal femur angle, MPTA–medial proximal tibia angle.

Radiological evaluation demonstrated no significant differences between the groups with regard to leg alignment and position of the femoral and tibial component but a tendency towards better alignment in the PSI group (Fig 5). A total of 80.9% in the PSI group and 65.7% in the conventional group were within three degrees deviation from a neutral leg axis (p = 0.055). This tendency was similar for the alignment of the femoral (91.2% vs 81.4%, p = 0.138) and tibial component (92.6% vs 84.3%, p = 0.183) in the coronal plane. However, only 72.1% of all whole-leg standing radiographs postoperatively were of good quality [15], which reduced the accuracy of the radiological analysis.

Both groups demonstrated significant improvement in pain, knee function and health-related quality of life. There were no differences at any point in time between the groups in the pain VAS, Oxford Knee Score and EQ VAS Health State (Table 3). Looking at the five dimensions separately, only “self care” demonstrated a difference (12% in the standard group vs 3% in the PSI group with “some problems”).

**Table 3:** Patient reported outcomes: Pain VAS, OKS and EQ VAS.

	PSI N = 67	Conventional N = 67	p-value
**Pain VAS (0–100 points)**			
** Preoperative**	63.5 (18.0)	68.6 (13.0)	0.062
** 6 weeks**	28.2 (21.2)	29.1 (21.1)	0.792
** 6 months**	15.7 (19.0)	19.1 (21.2)	0.332
** 12 months**	9.3 (15.9)	11.5 (18.6)	0.457
** Improvement**	54.3 (22.2)	57.1 (21.3)	0.444
**Oxford Knee Score (0–48 points)**			
** Preoperative**	22.0 (6.6)	21.2 (6.0)	0.477
** 6 weeks**	29.9 (7.5)	29.8 (7.0)	0.924
** 6 months**	39.1 (7.9)	37.1 (8.7)	0.184
** 12 months**	43.4 (5.4)	41.5 (7.9)	0.108
** Improvement**	21.4 (8.5)	20.3 (8.9)	0.457
**EQ-5D VAS Health State (0–100)**			
** Preoperative**	60.8 (18.9)	57.4 (20.3)	0.307
** 6 weeks**	71.0 (18.5)	68.4 (18.4)	0.416
** 6 months**	74.5 (19.0)	70.7 (19.1)	0.251
** 12 months**	77.0 (17.4)	71.0 (22.2)	0.085
** Improvement**	16.2 (23.8)	13.7 (24.6)	0.549

Mean cost reduction in the OR was 65 € of for one instrument tray and 216 € for 6 minutes in the OR. Taking in account the additional costs of MRI (300€) and the PSI cutting guides (450€), PSI caused in sum extra costs at a value of 469 €.

Taking the best-case scenario from center 2 with a reduction of 4 instrument trays and a reduction of 9 minutes OR time, there resulted extra costs of 166 € for PSI.

## Discussion

The use of patient-specific instrumentation resulted in a reduction of instrument trays and total time in the OR and a non-significant tendency towards better alignment. Knee pain and Oxford Knee Score were not different between patients treated with PSI or conventional instrumentation.

PSI was introduced to reduce OR time and improve implant alignment in TKA. Several studies have investigated implant alignment. In a recent meta-analysis of 44 studies [16] a significantly lower risk of mechanical axis malalignment outside a range of ± 3° was found. There were 20.2% outside a range of ± 3° from a neutral mechanical axis with PSI and 25.7% with conventional instrumentation. The proportion of knees with malalignment of more than 3° of the femoral / tibial component were 10.3% / 10.5% with PSI and 11.0% / 7.9% with conventional instrumentation. This is consistent with the results of the present study. The differences between the groups were even greater (femoral / tibial component outlier 8.8% / 7.4% for PSI vs 18.6% / 15.7% for conventional instrumentation) but did not reach statistical significance due to the limited number of knees. Additionally, it has been demonstrated that novice surgeons are more likely to replicate preoperative planning when using patient-specific instrumentation [17]. It might therefore be a valuable tool especially for low-volume surgeons to guarantee correct alignment.

In the above mentioned meta-analysis [16] the pooled operative time was reduced by only 4.4 minutes with PSI which is consistent with the present study. This minor reduction by itself does not justify the use of this costly technology. However, additional OR resources could be saved. On average, one instrument tray was reduced. It should be noted that in the individual centers the reduction of the instrument trays turned out very differently. The reduction of surgical trays seems to depend mainly on the organization and processes of the OR. While one center could not save any instrument trays, another center was able to reduce the number by four trays, which is consistent to the decrease of four surgical trays as reported in the meta-analysis by Huijbregts et al [18]. This reduction could be achieved in all centers by reorganizing OR processes when using PSI instrumentation as a standard in every case. However, PSI leads to additional costs for MRI and the cutting guides, without equally reducing costs in the OR. Similar results have been reported by Watters [11] and Barrack [7], in which studies the additional costs exceed the savings in OR costs and processing costs for the instrument trays. If all centers in this study would reduce the instrument trays as the most efficient center did, this would still result in extra costs of approximately 166 € per case. In addition, there is increased time required by the patient and the surgeon, for attending the MRI and extra planning and approving of the surgical plan for the bone cuts [8]. Furthermore, it is currently not possible to rely completely on PSI as in about 10% of the cases additional instruments were needed. Even if this rate might be reduced by constant use of PSI, there will still be the need for conventional instruments as a backup.

With regard to short-term functional outcome, no significant differences between the groups were shown in the Oxford Knee Score or pain VAS. These results are consistent to other studies [5, 6, 19].

Only few studies evaluated health-related quality of life in PSI but with different measurement instruments. Abdel et al. [20] observed no significant differences at 3 months using the SF-12, Chen et al. [4] detected significant better scores for PSI in the mental component subscale of the SF-36 at 6 months, which, however, were equalized 2 years after surgery. The EQ-5D was only reported by Boonen et al. [21], who reported no significant differences between the two groups after a mean follow-up of 44 months. The present study comes to the same result, comparing the 12-months-follow-up.

We acknowledge some limitations. Patients were not randomly assigned to one of the treatment options. However, patients were similar in both groups with regard to known factors, which influence the outcome after TKA. Only BMI was significantly lower by 2 kg/m^2^, which does not seem to be clinically relevant. There was no strictly standardized technique between the two groups and between the three centers. The organization and processes in the OR are different in each hospital as are the costs. The economic calculation is therefore only a rough estimate for hospitals in Germany. However, this reflects the reality and variation of health care as to our knowledge there is no German hospital, which exclusively uses patient specific instrumentation. Finally, planning for the PSI technology took about 10 minutes longer which was not included in the calculation because it took not place in OR.

In our setting MRI was required to create a 3D-model of the knee to plan implant position and size preoperatively and produce the PSI. CT-based PSI could be a potential cost saver as scan times are shorter and costs lower. However, it is also associated with lower accuracy for TKA regarding the coronal limb axis and increased radiation exposure compared to MRI-based PSI [22].

Future research should focus on making PSI cheaper and easier available. As 3D-printing technologies continue to evolve and are becoming more accessible, PSI may also become more affordable. Furthermore, it should be investigated if the tendency of more accurate implant position results in a reduced revision rate in the long-term.

## Conclusion

The use of PSI was associated with significantly less OR resources. However, the savings did not equal the costs for this technology. Even in the best-case scenario the cost of PSI TKA was 166 € higher than conventional TKA. There was no differences between both groups in the outcome after TKA with regard to implant position, leg axis, pain, function and health-related quality of life. PSI seems therefore useful in special cases in which conventional instrumentation is difficult or impossible. Further improvement is necessary to implement PSI as a standard for TKA.

## References

[1] Thiele K, Perka C, Matziolis G, Mayr HO, Sostheim M, Hube R. Current failure mechanisms after knee arthroplasty have changed: polyethylene wear is less common in revision surgery. J Bone Joint Surg Am. 2015;97(9):715–20. Epub 2015/05/08. doi: 10.2106/JBJS.M.01534 . 25948517

[2] Jones CW, Jerabek SA. Current Role of Computer Navigation in Total Knee Arthroplasty. J Arthroplasty. 2018;33(7):1989–93. Epub 2018/03/07. doi: 10.1016/j.arth.2018.01.027 . 29506932

[3] Pietsch M, Djahani O, Hochegger M, Plattner F, Hofmann S. Patient-specific total knee arthroplasty: the importance of planning by the surgeon. Knee Surg Sports Traumatol Arthrosc. 2013;21(10):2220–6. Epub 2013/08/15. doi: 10.1007/s00167-013-2624-7 . 23942881

[4] Chen JY, Chin PL, Tay DK, Chia SL, Lo NN, Yeo SJ. Functional Outcome and Quality of Life after Patient-Specific Instrumentation in Total Knee Arthroplasty. J Arthroplasty. 2015;30(10):1724–8. Epub 2015/05/06. doi: 10.1016/j.arth.2015.04.007 . 25937100

[5] Goyal T, Tripathy SK. Does Patient-Specific Instrumentations Improve Short-Term Functional Outcomes After Total Knee Arthroplasty? A Systematic Review and Meta-Analysis. J Arthroplasty. 2016;31(10):2173–80. doi: 10.1016/j.arth.2016.03.047 . 27129762

[6] Mannan A, Akinyooye D, Hossain F. A Meta-analysis of Functional Outcomes in Patient-Specific Instrumented Knee Arthroplasty. J Knee Surg. 2017;30(7):668–74. Epub 2016/12/03. doi: 10.1055/s-0036-1593869 . 27907935

[7] Barrack RL, Ruh EL, Williams BM, Ford AD, Foreman K, Nunley RM. Patient specific cutting blocks are currently of no proven value. J Bone Joint Surg Br. 2012;94(11 Suppl A):95–9. doi: 10.1302/0301-620X.94B11.30834 . 23118393

[8] Nunley RM, Ellison BS, Ruh EL, Williams BM, Foreman K, Ford AD, et al. Are patient-specific cutting blocks cost-effective for total knee arthroplasty? Clin Orthop Relat Res. 2012;470(3):889–94. doi: 10.1007/s11999-011-2221-3 ; PubMed Central PMCID: PMC3270159. 22183476 PMC3270159

[9] Slover JD, Rubash HE, Malchau H, Bosco JA. Cost-effectiveness analysis of custom total knee cutting blocks. J Arthroplasty. 2012;27(2):180–5. Epub 2011/06/17. doi: 10.1016/j.arth.2011.04.023 . 21676584

[10] Thienpont E, Paternostre F, Van Wymeersch C. The indirect cost of Patient-Specific Instruments. Acta Orthop Belg. 2015;81(3):462–70. Epub 2015/10/06. . 26435242

[11] Watters TS, Mather RC, Browne JA 3rd, Berend KR, Lombardi AV, Jr., Bolognesi MP. Analysis of procedure-related costs and proposed benefits of using patient-specific approach in total knee arthroplasty. J Surg Orthop Adv. 2011;20(2):112–6. Epub 2011/08/16. . 21838072

[12] Naal FD, Impellizzeri FM, Sieverding M, Loibl M, von Knoch F, Mannion AF, et al. The 12-item Oxford Knee Score: cross-cultural adaptation into German and assessment of its psychometric properties in patients with osteoarthritis of the knee. Osteoarthritis Cartilage. 2009;17(1):49–52. doi: 10.1016/j.joca.2008.05.017 . 18602843

[13] Greiner W, Claes C, Busschbach JJ, von der Schulenburg JM. Validating the EQ-5D with time trade off for the German population. Eur J Health Econ. 2005;6(2):124–30. doi: 10.1007/s10198-004-0264-z . 19787848

[14] Sim J, Waterfield J. Validity, reliability and responsiveness in the assessment of pain. Physiother Theory Pract. 1997;13(1):23–37.

[15] Dexel J, Kirschner S, Gunther KP, Lutzner J. Agreement between radiological and computer navigation measurement of lower limb alignment. Knee Surg Sports Traumatol Arthrosc. 2014;22(11):2721–7. Epub 2013/07/09. doi: 10.1007/s00167-013-2599-4 . 23832176

[16] Thienpont E, Schwab PE, Fennema P. Efficacy of Patient-Specific Instruments in Total Knee Arthroplasty: A Systematic Review and Meta-Analysis. J Bone Joint Surg Am. 2017;99(6):521–30. Epub 2017/03/16. doi: 10.2106/JBJS.16.00496 . 28291186

[17] Ng CTJ, Newman S, Harris S, Clarke S, Cobb J. Patient-specific instrumentation improves alignment of lateral unicompartmental knee replacements by novice surgeons. Int Orthop. 2017;41(7):1379–85. Epub 2017/05/14. doi: 10.1007/s00264-017-3468-4 . 28500496

[18] Huijbregts HJ, Khan RJ, Sorensen E, Fick DP, Haebich S. Patient-specific instrumentation does not improve radiographic alignment or clinical outcomes after total knee arthroplasty. Acta Orthop. 2016;87(4):386–94. doi: 10.1080/17453674.2016.1193799 ; PubMed Central PMCID: PMC4967282. 27249110 PMC4967282

[19] Predescu V, Prescura C, Olaru R, Savin L, Botez P, Deleanu B. Patient specific instrumentation versus conventional knee arthroplasty: comparative study. Int Orthop. 2017;41(7):1361–7. Epub 2016/12/21. doi: 10.1007/s00264-016-3356-3 . 27995304

[20] Abdel MP, Parratte S, Blanc G, Ollivier M, Pomero V, Viehweger E, et al. No benefit of patient-specific instrumentation in TKA on functional and gait outcomes: a randomized clinical trial. Clin Orthop Relat Res. 2014;472(8):2468–76. Epub 2014/03/08. doi: 10.1007/s11999-014-3544-7 ; PubMed Central PMCID: PMC4079860. 24604110 PMC4079860

[21] Boonen B, Schotanus MG, Kerens B, van der Weegen W, Hoekstra HJ, Kort NP. No difference in clinical outcome between patient-matched positioning guides and conventional instrumented total knee arthroplasty two years post-operatively: a multicentre, double-blind, randomised controlled trial. Bone Joint J. 2016;98-B(7):939–44. doi: 10.1302/0301-620X.98B7.37274 . 27365472

[22] Wu XD, Xiang BY, Schotanus MGM, Liu ZH, Chen Y, Huang W. CT- versus MRI-based patient-specific instrumentation for total knee arthroplasty: A systematic review and meta-analysis. Surg-J R Coll Surg E. 2017;15(6):336–48. doi: 10.1016/j.surge.2017.06.002 WOS:000418216700005. 28756064

